# Systemic Delivery of mPEG‐Masked Trispecific T‐Cell Nanoengagers in Synergy with STING Agonists Overcomes Immunotherapy Resistance in TNBC and Generates a Vaccination Effect

**DOI:** 10.1002/advs.202203523

**Published:** 2022-09-11

**Authors:** Ming Shen, Chuanrong Chen, Qianqian Guo, Quan Wang, Jinghan Liao, Liting Wang, Jian Yu, Man Xue, Yourong Duan, Jiali Zhang

**Affiliations:** ^1^ State Key Laboratory of Oncogenes and Related Genes Shanghai Cancer Institute Renji Hospital School of Medicine Shanghai Jiao Tong University Shanghai 200032 China; ^2^ Shanghai Institute for Biomedical and Pharmaceutical Technologies Shanghai 200032 China; ^3^ Department of Oncology Yijishan Hospital of Wannan Medical College Wuhu 240001 China; ^4^ State Key Laboratory of Oncogenes and Related Genes Renji Hospital School of Biomedical Engineering Shanghai Jiao Tong University Shanghai 200127 China

**Keywords:** cancer stem cells, PD‐L1 blockade, STING agonist, T‐cell engager, triple‐negative breast cancer

## Abstract

T‐cell engagers (TCEs) represent a breakthrough in hematological malignancy treatment but are vulnerable to antigen escape and lack a vaccination effect. The “immunologically cold” solid tumor presents substantial challenges due to intratumor heterogeneity and an immunosuppressive tumor microenvironment (TME). Here, a methoxy poly(ethylene glycol) (mPEG)‐masked CD44×PD‐L1/CD3 trispecific T‐cell nanoengager loaded with the STING agonist c‐di‐AMP (CDA) (PmTriTNE@CDA) for the treatment of triple‐negative breast cancer (TNBC) is rationally designed. PmTriTNE@CDA shows tumor‐specific accumulation and is preferentially unmasked in response to a weakly acidic TME to prevent on‐target off‐tumor toxicity. The unmasked CD44×PD‐L1/CD3 trispecific T‐cell nanoengager (TriTNE) targets dual tumor‐associated antigens (TAAs) to redirect CD8+ T cells for heterogeneous TNBC lysis while achieving PD‐L1 blockade. PmTriTNE synergized with CDA to transform the cold tumor into a hot tumor, eradicate the large established TNBC tumor, and induce protective immune memory in a 4T1 orthotopic tumor model without causing obvious toxicity. PmTriTNE@CDA shows potent efficacy in cell line‐derived xenograft (CDX) and patient‐derived xenograft (PDX) mouse models. This study serves as a proof‐of‐concept demonstration of a nanobased TCEs strategy to expand therapeutic combinations that previously could not be achieved due to systemic toxicity with the aim of overcoming TNBC heterogeneity and immunotherapy resistance.

## Graphical abstract

CD44×PD‐L1/CD3 trispecific T‐cell nanoengager loaded with the STING agonist c‐di‐AMP (CDA) (PmTriTNE@CDA) shows tumor‐specific accumulation and the preferentially unmasked in response to the weakly acidic TME to avoid on‐target off‐tumor toxicity. CDA synergizes with PmTriTNE to eliminate triple‐negative breast cancer (TNBC), especially cancer stem cells (CSCs) and coordinate innate and adaptive immunity to achieve whole cancer immunity cycle cascade amplification against heterogeneous TNBC and generate a vaccination effect.

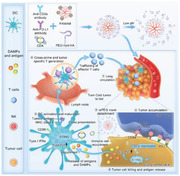

## 1 Introduction

Triple‐negative breast cancer (TNBC), which is categorized as the “immunologically cold” type, is characterized by low immunogenicity and an immunosuppressive tumor microenvironment (TME) and is enriched with cancer stem cells (CSCs).^[^ ^1^ ^]^ Despite the breakthrough of PD‐L1 immune checkpoint blockade (ICB) therapy for the clinical treatment of solid cancers, promotion of the T‐cell‐mediated destruction of TNBC remains a challenge due to the lack of endogenous T cells that recognize tumor cells through T‐cell receptors (TCRs).^[^ ^2^ ^]^ CSCs, which contribute to TNBC heterogeneity and drive therapy resistance, downregulate MHC expression and favor immune surveillance evasion. A recent study found that ICB therapy selectively enriched CSCs, which led to therapy resistance and tumor recurrence.^[^ ^3^ ^]^ T‐cell engagers (TCEs), such as bispecific T‐cell engagers (BiTEs), target both CD3 on T cells and a tumor‐associated antigen (TAA) on tumor cells to form an immunological cytolytic synapse between target cancer cells and T cells and lead to target cell lysis independent of TCR specificity, which is promising for the targeting of poorly immunogenic tumors.^[^ ^4^ ^]^ However, the lack of TAA that exclusively expressed in tumor cells, TNBC heterogeneity and an immunosuppressive TME hinder the application of TCEs.^[^ ^4^ ^]^

TCEs rely uniquely on TAA expressed on target cells to redirect T cells, and the presence of intratumorally heterogeneous TAAs in solid tumors makes the selection of the appropriate target difficult. Moreover, the downregulation of TAA or the emergence of preexisting TAA‐null variants on tumor cells lead to tumor escape.^[^ ^5^ ^]^ As observed in recent studies, simultaneously targeting multiple TAAs can increase the TCE tumor selectivity and binding affinity to overcome the heterogeneity of TAAs.^[^ ^5^ ^]^ CD44 and PD‐L1 are overexpressed in heterogeneous TNBC populations, especially by CSCs, and play a key role in facilitating immune evasion and maintaining CSC properties.^[^ ^6^ ^]^ These molecules could be used as TAAs to target heterogeneous TNBC populations while avoiding the immune selection of TAA‐deficient tumor clones.^[^ ^7^ ^]^ However, similar to most TAAs, CD44 and PD‐L1 are also expressed on normal tissues, and the systemic administration of TCEs might cause intolerable on‐target off‐tumor toxicity.^[^ ^8^ ^]^ Thus, the development of TCE strategies that specifically kill TNBC tumors without damaging normal tissues is urgently needed.

The immunosuppressive TME, which exhibits features including the lack of sufficient preexisting tumor‐infiltrating lymphocytes (TILs), the intrinsic suppression of type I IFN signaling, and the low immunogenicity of CSCs, leads to TCE therapy resistance and difficulties in generating long‐term antitumor immunity.^[^ ^9^ ^]^ Innate immune cells, particularly dendritic cells (DCs), participate in all steps of T‐cell generation and activity against tumor cells, which is essential to the onset of tumor‐specific T‐cell responses and the development of protective memory T cells.^[^ ^10^ ^]^ The stimulator of interferon genes (STING) signaling pathway is a critical link between innate and adaptive immunity in the TME. Recent research has demonstrated that the local delivery of STING agonists, such as cyclic dinucleotides (CDNs), upregulates type I interferons (IFNs) in the TME and elicits robust anticancer efficacy in combination with PD‐L1 blockade therapy.^[^ ^11^ ^]^ Type I IFN can reverse TNBC CSC properties and increase tumor immunogenicity while promoting DC maturation and recruiting natural killer (NK) and CD8+ T cells into the TME to promote antitumor immunity.^[^ ^9^, ^12^ ^]^ Thus, exploring whether STING agonists can also act synergistically with TCEs to transform “immune‐cold” tumors into “immune‐hot” tumors and achieve T‐cell‐based long‐term immune memory to overcome TNBC would be interesting. However, the short half‐life of CDNs and TCEs in vivo and systemic administration of either CDNs or TCEs may cause unacceptable on‐target off‐tumor immune‐related adverse events (iRAEs) in patients because both CDNs and TCEs cannot be selectively enriched at the tumor site.^[^ ^13^ ^]^ Thus, a novel combinational strategy is needed to extend their half‐lives and tumor‐specific targeting ability.

**Scheme 1:**
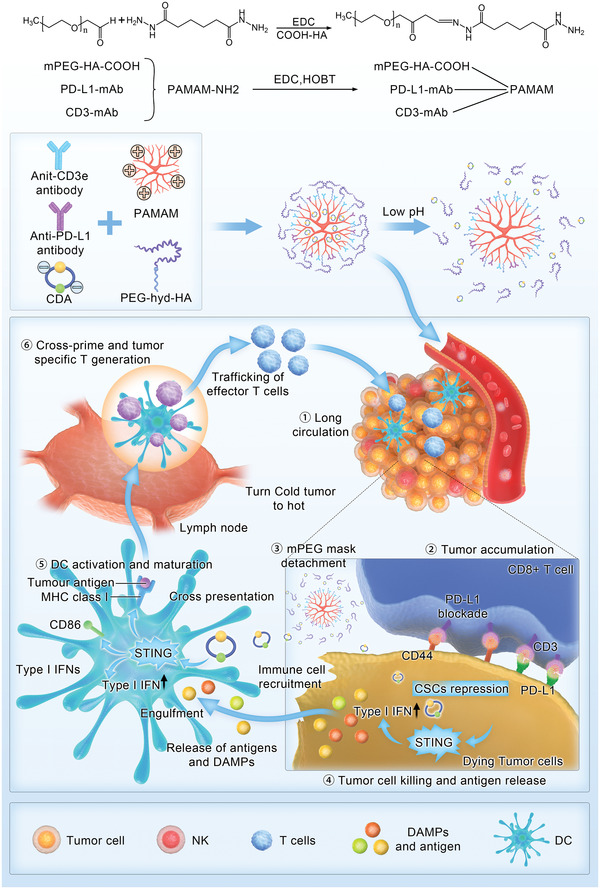
PmTriTNE@CDA elicited long‐lasting, multilayered TNBC control. CDA could intrinsically repress CSC properties and increase tumor immunogenicity while extrinsically promoting DC maturation/antigen cross‐priming and the local recruitment/activation of T cells and NK cells. Unmasked TriTNE can block PD‐1/PD‐L1 ligation and redirect CD8+ T cells to kill CD44‐ and PD‐L1‐high tumor cells, including CSCs. The dead tumor cells then release DAMPs and tumor antigen, which enable tumor antigen spread to achieve whole cancer immunity cycle cascade amplification against heterogeneous TNBC and generate a vaccination effect.

Methoxy poly(ethylene glycol) (mPEG) nanoparticles (NPs) can act as multifunctional platforms to integrate protein therapeutics and small nucleic acid drugs, prolong their circulation time, facilitate their passive enrichment at the tumor site and enable a TME‐specific response, which are effects that cannot be easily achieved using traditional techniques.^[^ ^14^ ^]^ Conventional nanomedicines have a size of ≈100 nm. However, for immune synapse (IS) formation, which is a prerequisite for TCE‐mediated target cell killing, the distance between T cells and tumor cells needs to be within ≈15 nm.^[^ ^15^ ^]^ The use of NPs that are too large can easily cause the distance between T cells and tumor cells to be greater than 35 nm, which will prevent the formation of IS.^[^ ^16^ ^]^ Moreover, the IS clears and excludes molecules greater than 35 nm in size.^[^ ^17^ ^]^ Thus, ultrasmall NPs (≈15 nm) are most suitable for the development of TCE and may better penetrate the extracellular matrix (ECM) of solid tumors than conventional NPs. Poly(amidoamine) (PAMAM) is a single molecular entity with a small size (≈4 nm) that possesses an adjustable positive charge and many cavities for the loading of active ingredients.^[^ ^18^ ^]^ Thus, we adopted a modular design approach using PAMAM dendrimers as substrates to develop a weakly acidic TME‐responsive mPEG‐masked CD44×PD‐L1/CD3 trispecific T‐cell nanoengager (PmTriTNE) that encapsulates a STING agonist (c‐di‐AMP [CDA]) (**Scheme** **1**). An mPEG mask with a hydrazone bond (hyd) linker on oligomeric hyaluronic acid (o‐HA) enables the prolonged circulation of NPs and works as a spatial shield for blocking the antigen binding ability of CD44×PD‐L1/CD3 trispecific T‐cell nanoengager (TriTNE) to avoid off‐target side effects. The Hyd linker is broken in the weakly acidic TME, which leads to mPEG shedding and the rapid release of STING and exposes o‐HA, anti‐CD3 antibody (ɑ‐CD3) and anti‐PD‐L1 (ɑ‐PD‐L1) on TriTNE. The unmasked TriTNE simultaneously targets PD‐L1 and CD44 on tumors to redirect T cells to kill heterogeneous TNBC tumors, including CSCs, while TriTNE can also reverse CD8+ T‐cell immunosuppression by blocking PD‐1/PD‐L1 ligation. Moreover, CDA synergizes with PmTriTNE to inhibit CSCs and coordinate innate and adaptive immunity to eradicate TNBC and induce long‐term protective immune memory in a mouse model with syngeneic 4T1 orthotopic tumors without obvious systemic toxicity. Moreover, PmTriTNE@CDA, which targets human PD‐L1 and CD3, showed robust antitumor efficacy in both cell line‐derived xenograft (CDX) and patient‐derived xenograft (PDX) models, which indicated that PmTriTNE@CDA has potential for clinical translation. In summary, rationally designed PmTriTNE@CDA enables novel therapeutic combinations that were previously inaccessible due to systemic toxicity and can be used to harness both innate and adaptive immunity to eradicate heterogeneous TNBC and elicit a vaccination effect.

## 2 Results

### 2.1 Generation and Characterization of pH‐Responsive PmTriTNE@CDA

**Figure 1:**
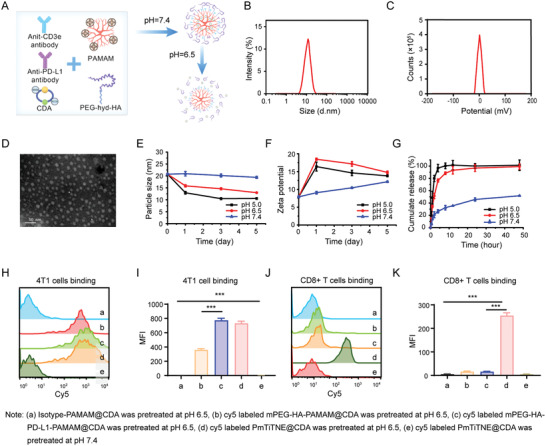
A tumor microenvironment‐responsive nanoplatform for tumor‐specific combinational immune therapy. A) Schematic showing the generation of PmTriTNE@CDA. B) Diameter of PmTriTNE@CDA. C) *Z*‐potential of PmTriTNE@CDA. D) Morphology of PmTriTNE@CDA. E) The particle size changed under the indicated pH values. F) The *Z*‐potential changed under the indicated pH values. G) Characterization of CDA release under the indicated pH values. H–K) 4T1 cells or CD8+ T cells were incubated with cy5‐labeled *α*‐Isotype‐PAMAM@CDA pretreated with pH 6.5 buffer, cy5‐labeled mPEG‐HA‐PAMAM@CDA pretreated with pH 6.5 buffer (ii), cy5‐labeled mPEG‐HA‐PD‐L1‐PAMAM@CDA pretreated with pH 6.5 buffer (iii), cy5‐labeled PmTiTNE@CDA pretreated with pH 6.5 buffer (iv) or cy5‐labeled PmTiTNE@CDA pretreated with pH 7.4 buffer (v) for 20 min at 4 °C. Then, flow cytometry analysis of the specific binding of PmTriTNE to 4T1 cells (H,I) and CD8+ T cells (J,K). The experimental results are shown as the means ± SDs (*n* = 3). *p*‐values are calculated using one‐way ANOVA with Tukey's multiple comparison. ***p < 0.001.

Most clinically approved nanomedicines have a size of ≈100 nm to allow the passive targeting of tumors, and mPEGylation endows nanomedicines with a long circulation time and enhanced penetration residence (EPR) ability.^[^ ^19^ ^]^ However, the enhanced penetration residence (EPR) effect in the clinic is limited due to the ECM barrier in solid tumors and the lack of deep tumor penetration.^[^ ^20^ ^]^ Small NPs with a diameter less than 30 nm reportedly show a better ability to overcome the ECM than larger particles and enable deep tumor penetration.^[^ ^21^ ^]^ Most importantly, particles larger than 30 nm compromise IS formation and are rapidly excluded from the IS.^[^ ^17^ ^]^ Thus, a small size is crucial for the development of a successful T‐cell nanoengager. As illustrated in **Figure** **1**A, we fabricated PmTriTNE@CDA using PAMAM, a single molecular entity of ≈4 nm, as a flexible platform to anchor mPEG‐hyd‐HA, PD‐L1 and CD3 and load CDA by electrostatic adsorption. The synthetic route and ^1^H NMR spectra of PmTriTNE are shown in Figure S1, Supporting Information. The diameter, *Z*‐potential and morphology of PmTriTNE@CDA are shown in Figure 1B–D. The particle size and potential were stable at pH 7.4, indicating that PmTriTNE was stable at physiological pH in vivo. A slow increase in potential was observed in PBS at pH 7.4, and this increase was caused by the slow release of CDA. In PBS at pH 6.5, the particle size decreased over time, and this decrease might have been caused by hydrazone bond breakage and the exposure of negatively charged o‐HA (Figure 1E,F). The rise in potential was likely due to the rapid release of CDA. The assessment of particle size and potential demonstrated that CDA and mPEG could detach in an acidic environment. CDA was released slowly at pH 7.4, and only ≈50% was released after 48 h. In an acidic medium, the release of CDA was significantly accelerated. Approximately 80% of CDA was released within 2 h, and more than 95% was released within 4 h in PBS at pH 6.5 (Figure 1G). PmTriTNE showed pH‐sensitive release characteristics, which enabled as little CDA as possible to be released into the blood circulation but as much CDA as possible to be released rapidly when PmTriTNE reached the tumor tissue. Therefore, CDA could exert curative effects without systemic toxicity. Thus, these results suggest that when the NPs arrive at the tumor site, CDA is released rapidly, and the targeting antibodies are exposed to act as a bridge to redirect T cells to kill targeted tumor cells.

To measure the ratio of ɑ‐PD‐L1 and ɑ‐CD3 in the final particles, the TriTNE were prepared with FITC‐ɑ‐PD‐L1 and APC‐ɑ‐CD3. The fluorescence intensity of FITC‐ɑ‐PD‐L1 and APC‐ɑ‐CD3 under a series of concentration gradients were measured by the microplate reader, respectively. The fluorescence intensity of FITC and APC of the PmTriTNE was measured and the ɑ‐PD‐L1:ɑ‐CD3 ratio was by calculation based on the respective standard curves (Figure S2, Supporting Information). We found that the ɑ‐PD‐L1:ɑ‐CD3 ratio on the NP was 1:0.89, which was close to 1:1. Then, we further examined the antigen targeting ability of cy5‐labeled PmTriTNE@CDA pretreated at pH 7.4 or 6.5 to 4T1 cells and CD8+ T cells to further confirm the functionality of the pH‐responsive mPEG mask. By flow cytometry analysis, we confirmed that PmTriTNE@CDA pretreated at pH 6.5 but not PmTriTNE@CDA pretreated at pH 7.4 could specifically bind 4T1 cells (CD44+PD‐L1+) and CD8+ T cells (CD3+) (Figure 1H–K, and Figures S3 and S4, Supporting Information), which indicated that mPEG could mask the antigen binding ability of TriTNE at pH 7.4 while specifically unmasking at pH 6.5. We measured the expression level of PD‐L1 and CD44 on 4T1 cells and found that most of the 4T1 cells being PD‐L1 and CD44 double‐positive cells (Figure S5, Supporting Information). Moreover, PmTriTNE@CDA pretreated at pH 6.5 showed higher levels of tumor cell binding than mPEG‐HA‐PAMAM@CDA pretreated at pH 6.5 and mPEG‐HA‐PD‐L1‐PAMAM@CDA pretreated at pH 6.5, which suggested that dual targeting could increase the binding affinity and selectivity (Figure 1H,I). These data confirmed that mPEG with a hydrazone linker acts as a universal mask to block the antibody and o‐HA of TriTNE binding to antigen while enabling preferential unmasking, target binding, and CDA release in the unique weakly acidic TME. On the one hand, mPEG masking extends the half‐life and sterically blocks target antigen engagement with low immunogenicity, and on the other hand, unmasked TriTNE binds to dual tumor antigens to overcome TAA heterogeneity and PD‐L1‐mediated immune escape.

### 2.2 TriTNE Redirected and Activated CD8+ T Cells to Specifically Kill 4T1 Cells

**Figure 2:**
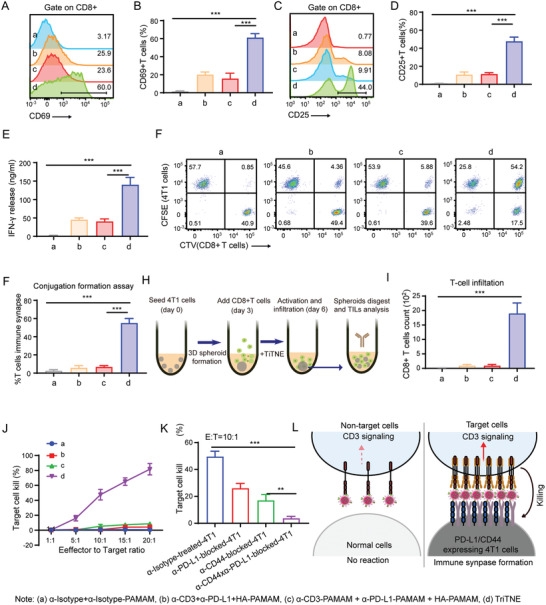
TriTNE redirected T cells to kill 4T1 cells in vitro. The 4T1 cells were cultured for 1 day, and CD8+ T cells were then cultured in the presence of the *α*‐Isotype+*α*‐Isotype‐PAMAM (a), *α*‐CD3+*α*‐PD‐L1+HA‐PAMAM (b), *α*‐CD3‐PAMAM + *α*‐PD‐L1‐PAMAM + HA‐PAMAM (c) or TriTNE (d) for 24 h. Subsequently, the expression levels of CD69 (A,B) or CD25 (C,D) on T cells and IFN‐*γ* production were measured (E). F) CFSE‐labeled 4T1 cells CTV‐labeled CD8+ T cells were mixed with CFSE‐labeled 4T1 cells and then incubated in the presence of the indicated NPs. The immune synapse formation (CFSE+CTV+) population was then analyzed by flow cytometry. G) The statistical analysis of immune synapse formation from (E). H) 4T1 cell spheroids were formed for 72 h, and then CD8+ T cells and TriTNE were added. After coculture for 3 days, the spheroids were collected and digested to analyze the tumor spheroid‐infiltrated T cells. I) Absolute number of CD8+ T cells that infiltrated breast cancer spheroids. J) TDCC was evaluated at the indicated E:T ratio, and cytotoxicity was determined by measuring the release of lactate dehydrogenase (LDH). K) 4T1 cells were preliminarily blocked by anti‐CD44 and anti‐PD‐L1 antibodies, and the TDCC of TriTNE was then evaluated with a 10:1 E:T ratio. L) Working model of the TriTNE mechanism in TAA‐positive tumor cells and TAA‐negative normal cells. The data are presented as the means ± SDs (*n*  =  3). *p*‐values are calculated using one‐way ANOVA with Tukey's multiple comparison. ***p < 0.001.

Since the mPEG mask could block the antigen binding ability of TriTNE and enable preferential unmasking in weakly acidic conditions, we further explored the antitumor function of TriTNE in vitro. Through CD8+ T‐cell and 4T1 coculture assays, we further explored the antitumor function of TriTNE and compared it with that achieved using combined treatment with single specific antibodies or NPs. CD25 and CD69 expression on the surface of T cells (**Figure** **2**A–D), as well as IFN‐*γ* production (Figure 2E), were most significantly increased in the TriTNE‐treated group, whereas that in the group subjected to combination treatment with single specific antibodies or NPs did not, indicating that TriTNE led to significantly higher levels of T‐cell activation than the combined treatment with single specific antibodies or NPs. We further explored whether TriTNE could efficiently induce IS formation, which is critical for T‐cell activation and tumor cell‐specific killing.^[^ ^22^ ^]^ We found that TriTNE could induce ≈50% IS formation, whereas the combined treatment with single specific antibodies or NPs could not (Figure 2F,G). The gating strategy of the IS formation population is as in Figure S6, Supporting Information. These data could explain why TriTNE could induce tumor‐specific T‐cell activation and why the single CD3‐targeting NPs could not efficiently activate T cells. To further evaluate the efficacy of TriTNE against solid tumors, a 3D tumor spheroid culture model was used to quantify TILs after TriTNE treatment (Figure 2H). We found that TriTNE treatment led to significantly higher levels of TIL infiltration than the combined treatment with single specific antibodies or NPs (Figure 2I).

Next, we assessed T‐cell‐dependent cellular cytotoxicity (TDCC) and found that TriTNE enabled T‐cell‐specific tumor cell killing and that TDCC increased as the effector cell (CD8+ T cell):target cell (4T1) ratio (E/T ratio) increased (Figure 2J). We used 4T1 cells with CD44/PD‐L1 dual blockade or single blockade as controls to further confirm whether the specificity of TriTNE depended on CD44 and PD‐L1 expression on tumor cells. Using the cell killing assay, we observed that the 4T1 cells with PD‐L1/CD44 blockade were immune to TriTNE‐mediated tumor killing and that the use of single CD44 or PD‐L1 blockade led to significantly lower levels of TriTNE‐mediated tumor cell killing than the use of isotype antibody in treated 4T1 cells (Figure 2K). These data suggest that TriTNE induced antigen‐dependent cytotoxicity in 4T1 cells and that both CD44 and PD‐L1 were required for optimal TriTNE‐mediated tumor cell killing.

CSCs contribute to intratumor heterogeneity and immunotherapy resistance. However, targeted immunotherapy against CSCs in TNBC is lacking. CD44 and PD‐L1 are overexpressed in heterogeneous TNBC populations and play key roles in CSC maintenance and immune escape.^[^ ^6^, ^23^ ^]^ Moreover, studies have indicated that the CSCs in TNBC exhibit higher CD44 and PD‐L1 expression levels than those in differentiated TNBC.^[^ ^24^ ^]^ Thus, we further explored whether TriTNE could efficiently eliminate CSCs. Aldehyde dehydrogenase (ALDH) is enriched in TNBC CSCs. TriTNE treatment led to a lower percentage of aldehyde dehydrogenase (ALDH) cells (Figure S7A,B, Supporting Information) as well as a lower ability to form TNBC spheres than the combined treatment with single specific antibodies or NPs (Figure S8, Supporting Information), indicating that TriTNE could efficiently eliminate CSCs among TNBC cells.

Overall, our results suggest that TriTNE exerted antitumor activity only in the context of PD‐L1/CD44 engagement and could efficiently eliminate CSCs. The working model of TriTNE is shown in Figure 2L. For nontarget cells that do not express PD‐L1 or CD44, no IS formation was induced by TriTNE, and T‐cell‐mediated cell lysis was not induced, while TriTNE was bound to both PD‐L1 and CD44 on target cells and CD3 on T cells, which enabled IS formation and T‐cell‐mediated target cell‐specific lysis. Therefore, TriTNE could redirect CD8 T cells to specifically kill target cells.

### 2.3 Systemic Administration of PmTriTNE@CDA Shows No Apparent Toxicity

**Figure 3:**
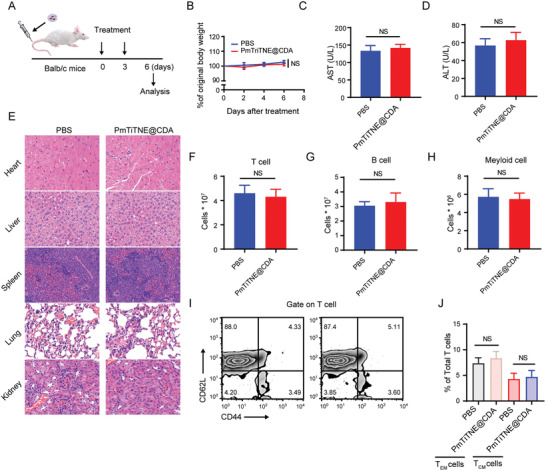
Absence of systemic toxicity following PmTriTNE@CDA treatment. A) BALB/c mice were treated with PBS or PmTriTNE@CDA and analyzed 3 days after the last treatment. B) Relative changes in mouse weight were examined during the treatment period. C,D) Serum ALT (C) and AST (D) levels were measured. E) Histological analysis of liver, spleen, heart, kidney, and lung tissues. F–H) The T‐cell (F), B‐cell (G), and myeloid cell (H) populations in the spleen were measured by flow cytometry. I) The T_EM_ and T_CM_ cell populations in the spleen were measured by flow cytometry. J) Statistical analysis of TEM and T_CM_ proportions among T cells from (I). Experimental results are shown as the mean ± SD (*n* = 5). Student's *t*‐test was used for comparisons of 2 groups and one‐way ANOVA with Tukey's multiple comparison was used for comparisons of more than 2 groups. NS, not significant.

TCEs show promising efficacy in inducing the remission of hematological tumors. However, their application to the treatment of solid cancers is challenging because of their toxicity to normal cells in healthy tissues that exhibit TAA expression. As STING is expressed on most cells, the systemic administration of CDA also shows severe systemic toxicity, which makes the use of combined treatment with TCEs and CDA unacceptable.^[^ ^25^ ^]^ Thus, we rationally designed PmTriTNE to exploit the intrinsically weak acidic TME to preferentially unmask and activate TriTNE in tumor tissues over healthy tissues, thereby mitigating off‐tumor toxicity. The safety of PmTriTNE upon systemic administration was assessed. Immune‐competent mice were intravenously injected with PmTriTNE@CDA or PBS, as illustrated in **Figure** **3**A. Treatment with PmTriTNE@CDA did not lead to a lower mouse weight than PBS treatment (Figure 3B). There was no significant increase in serum alanine aminotransferase (ALT) or aspartate aminotransferase (AST) levels, which are hallmarks of liver damage (Figure 3C,D). H&E staining of the major organs also confirmed that PmTriTNE@CDA caused no apparent systemic toxicity (Figure 3E). To further confirm the safety of PmTriTNE@CDA in vivo, we analyzed the immune cell populations in the spleen and CD8+ T‐cell subtypes. PmTriTNE@CDA did not alter the absolute number of T cells, B cells, NK cells, or myeloid cells in the spleen (Figure 3F–H). Moreover, the percentage of effect‐memory T (T_EM_) cells as well as central‐memory T (T_CM_) cells among T cells showed no significant difference between the two groups (Figure 3I,J). In summary, PmTriTNE@CDA treatment showed no obvious systemic toxicity and did not perturb immune homeostasis of spleen immune cells in vivo. Thus, mPEG‐masked PAMAM could act as a modular nanoplatform to achieve therapeutic combinations that previously could not be achieved due to unacceptable toxicity, making new treatment options possible.

### 2.4 PmTriTNE@CDA Eliminated Large Established Tumors in a 4T1 Orthotopic Mouse Model and Showed No Apparent Toxicity

**Figure 4:**
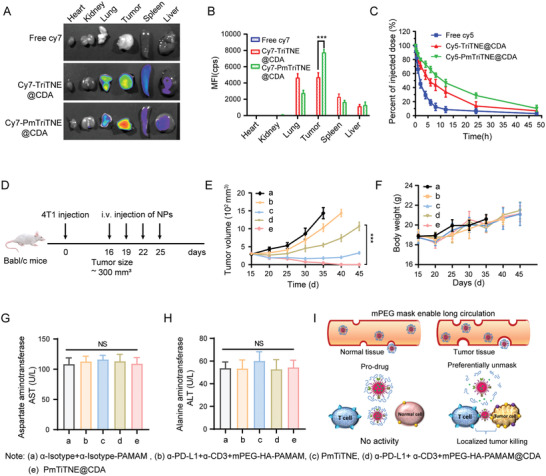
PmTriTNE@CDA generated superior antitumor efficacy in an orthotopic TNBC mouse model. A) Images of key organs and tumors 48 h after cy7, cy7‐TriTNE@CDA, or cy7‐PmTriTNE@CDA intravenous injection. B) Statistical analysis of the fluorescence intensities in (A). C) Pharmacokinetics of cy5‐PmTriTNE@CDA. D) Schematic diagram of the experimental design for TNBC treatment in 4T1 tumor‐bearing mice. Mice were intravenously administered *α*‐Isotype+*α*‐Isotype‐PAMAM (control) (i), ɑ‐PD‐L1+ ɑ‐CD3+mPEG‐HA‐ PAMAM (ii), PmTiTNE (iii), ɑ‐PD‐L1+ ɑ‐CD3+mPEG‐HA‐PAMAM@CDA (iv), or PmTiTNE@CDA (v). E) Curves showing tumor growth in 4T1 tumor‐bearing mice. F) Change in mouse weight during the treatment period. G,H) Serum AST (G) and ALT (H) levels at day 30. I) Diagram showing the in vivo working model of PmTriTNE@CDA. Experimental results are shown as the mean ± SD (*n* = 5). *p*‐values are calculated using one‐way ANOVA with Tukey's multiple comparison. ***p < 0.001.

Most nanomedicines have a size of ≈100 nm, and their accumulation at the tumor site in the clinic is limited by the ECM barrier in solid tumors.^[^ ^26^ ^]^ We hypothesized that the small PmTriTNE@CDA (≈30 nm) could better overcome the ECM barrier and that the preferentially unmasked ultrasmall TriTNE (≈15 nm) in the TME would more easily penetrate the solid tumor. To verify these hypotheses, we explored the tumor targeting and therapeutic efficacy of PmTriTNE@CDA in an orthotopic TNBC mouse model. According to the in vivo biodistribution data, PmTriTNE@CDA could more efficiently accumulate at the tumor site than the control (**Figure** **4**A,B). Moreover, cy5‐PmTriTNE@CDA had a longer circulation than free cy5 and cy5‐TriTNE@CDA (Figure 4C), which indicated that the mPEG mask could increase the half‐life of TriTNE.

We further explored the therapeutic efficacy and safety of PmTriTNE@CDA in an orthotopic 4T1 tumor mouse model, as illustrated in Figure 4D. We found that PmTriTNE led to significantly inhibited tumor growth, unlike PBS treatment (Figure 4E). However, combined treatment with single specific antibodies and NPs exerted antitumor effects only at an early stage, and tumor relapse eventually occurred, which indicated that PmTriTNE could prevent solid tumor immunotherapy resistance, probably due to enhanced tumor binding by targeting dual TAAs and the synergistic effects of both PD‐L1 blockade and T‐cell redirection. Moreover, PmTriTNE@CDA completely eradicated tumors. We also found that neither the weight of the mice (Figure 4F) nor the levels of ALT and AST (Figure 4G,H) in the NP‐treated groups were significantly different from those in the mice in the PBS control group, which indicated that TriTNE@CDA was efficacious in eradicating TNBC without causing obvious toxicity. Overall, PmTriTNE@CDA showed a unique functionality that triggered a specific antitumor response against TNBC, which was impossible to accomplish by a conventional combination treatment. As illustrated in Figure 4I, we hypothesize that preferential mPEG unmasking in response to the weakly acidic TME can rapidly release a STING agonist and activate TriTNE to optimize synapse formation and enable local tumor cell killing while minimizing systemic toxicity.

### 2.5 PmTriTNE@CDA Coordinated Innate Immunity and Adaptive Immunity to Achieve Long‐Lasting, Multilayered TNBC Control

**Figure 5:**
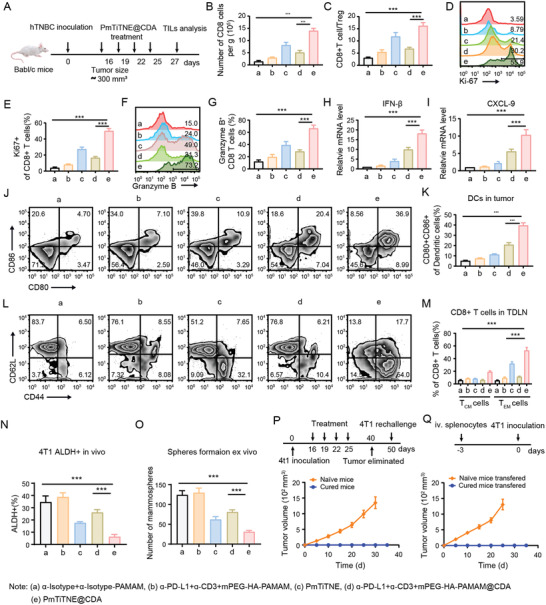
PmTriTNE@CDA transformed cold tumors into hot tumors and achieved long‐term immune memory against TNBC. A) BALB/c mice were inoculated with 4T1 tumor cells, and the tumor‐bearing mice were intravenously administered *α*‐Isotype+*α*‐Isotype‐PAMAM NPs (control) (i), ɑ‐PD‐L1+ɑ‐CD3 +mPEG‐HA‐PAMAM (ii), PmTiTNE (iii), ɑ‐PD‐L1+ɑ‐CD3+mPEG‐HA‐PAMAM@CDA (iv) or PmTiTNE@CDA (v) at the indicated time points. At day 27, TILs were measured by flow cytometry. B) CD8+ TIL absolute number. C) CD8+ TIL/Treg ratio. D) Expression of Ki67 in CD8+ TILs. E) Statistical analysis of the data in (D). F) Expression of granzyme B in CD8+ TILs. G) Statistical analysis of the data in (F). H,I) mRNA expression of IFN‐*β* (H) and CXCL9 (I) in tumor tissue. J) Maturation of DCs. K) Statistical analysis of the data in (J). L) Percentage of T_EM_s and T_CM_s among CD8+ T cells in TDLNs. M) Statistical analysis of the data in (L). N) ALDH expression in 4T1 tumors was measured by flow cytometry. O) Tumor masses were excised and processed for ex vivo primary mammosphere‐forming assays. P) BALB/c mice were treated with PmTriTNE@CDA on days 15, 18, and 21 after tumor implantation. Fifty days after treatment, the cured mice were rechallenged with 4T1 tumor cells, and naïve mice were used as a control. Then, tumor growth was monitored. Q) A total of 2 × 10^7^ splenocytes from cured mice or naïve mice were intravenously administered to 4T1 cell‐inoculated NSG mice. After 3 days, 4T1 cells were inoculated, and tumor growth was monitored. The data are presented as the means ± SDs (*n*  =  5). *p*‐values are calculated using one‐way ANOVA with Tukey's multiple comparison. ***p < 0.001.

We explored the mechanisms underlying the therapeutic effects of mTriTNE by examining TILs and innate immune cells within the TNBC mouse model (**Figure** **5**A). The TIL gating strategy is shown in Figure S9, Supporting Information. Impressively, the absolute number of CD8+ TILs (Figure 5B) and the CD8+ TIL/Treg ratio (Figure 5C) were significantly higher in the PmTriTNE and PmTriTNE@CDA groups than in the groups treated with PBS or the combined treatment with single specific antibodies or NPs. We further evaluated the expression of the proliferation marker Ki67 and the cytolysis factor granzyme B in CD8+ TILs and found that CD8+ TILs in the PmTriTNE and PmTriTNE@CDA groups exhibited a significantly higher percentage of Ki67+ CD8+ TILs and produced more granzyme B than those in the other groups (Figure 5D–G). Thus, PmTriTNE showed unique functionality by engaging T cells and tumor cells while simultaneously blocking PD‐L1 to efficiently activate and expand CD8+ TILs and thus elicit a potent antitumor response in the TME. Because CDA treatment can mobilize innate immune cells to boost TIL antitumor function, we hypothesized that CDA could coordinate with TriTNE to further promote CD8+ TIL‐mediated antitumor immunity, and the data suggested that the mTriTNE@CDA group had the highest number of TILs and the highest granzyme B expression.

The activation of STING in tumor cells and DCs upregulates type I IFNs and interferon‐stimulated genes (ISGs), which promotes DC maturation and antigen cross‐priming while recruiting CD8+ T cells and NKs to the tumor site.^[^ ^27^ ^]^ Moreover, ISGs can modulate antigen processing/presentation machinery (APM) to increase tumor immunogenicity.^[^ ^27^, ^28^ ^]^ Thus, we examined the expression of ISGs, including IFN‐*β* and CXCL9, in the tumor tissue and found that the PmTriTNE@CDA‐treated group exhibited the highest ISG expression levels (Figure 5H,I). Consistent with a previous report that demonstrated that ISGs can recruit both T cells and NK cells into the tumor site, the absolute number of NK cells was most significantly increased in the PmTriTNE@CDA‐treated group (Figure S10, Supporting Information). In addition, PmTriTNE@CDA treatment resulted in the highest proportion of mature DCs (Figure 5J,K). The gating strategy of DCs is as shown in Figure S11, Supporting Information. Mature DCs can migrate into the tumor drain lymph node (TDLN) to prime and activate tumor‐specific T cells.^[^ ^29^ ^]^ By examining T_EM_ and T_CM_ populations among CD8+ T cells in TDLNs, we found that the T_CM_ and T_EM_ proportions among CD8+ T cells in the PmTriTNE@CDA groups were highest of all the tested groups (Figure 5L,M), indicating that PmTriTNE@CDA could better coordinate adaptive and innate immune responses to fight cancer than the combination treatment groups. The gating strategy of the CD8+ T cells in TDLN is as shown in Figure S12, Supporting Information. ALDH^high^ CSC levels were found to be higher in TNBC tissues and cell lines than in tissue obtained from other breast cancer subtypes.^[^ ^30^ ^]^ Many studies have found that CD44 and PD‐L1 exhibit higher expression levels in CSCs than in differentiated TNBC;^[^ ^24^ ^]^ thus, PmTriTNE might better target CSCs, and we have shown that TriTNE could efficiently eliminate CSCs in vitro. Moreover, IFN‐*β* can suppress CSC properties in TNBC.^[^ ^9a^ ^]^ Thus, we explored whether PmTriTNE and CDA could exert synergistic effects on inhibiting CSCs. The gating strategy of ALDH+ 4T1 cells is as shown in Figure S13, Supporting Information. The PmTriTNE@CDA‐treated group showed the lowest ALDH+ cell populations (Figure 5N and Figure S14, Supporting Information) and ex vivo mammosphere formation (Figure 5O), which confirmed the anti‐CSC effect of PmTriTNE@CDA.

The key to successful immune treatment is the establishment of long‐term protective immunity against cancer, but TCEs exert their antitumor function mainly by relying on TAA to redirect polyclonal T cells to kill target cells and are inefficient in inducing the formation of long‐lasting antitumor‐specific memory T cells.^[^ ^31^ ^]^ Based on the data above, we hypothesized that PmTriTNE@CDA could redirect and expand CD8+ T cells while simultaneously reversing PD‐L1‐mediated immune suppression to kill tumor cells. Tumor antigens and DAMPs released from dead tumor cells then cooperate with CDA to promote DC maturation and cross‐priming, which enables tumor antigen spread and achieves in situ vaccine effects. To verify this hypothesis, we rechallenged cured mice with 4T1 cells on day 50 after treatment and monitored tumor growth and found that the tumors did not grow in the cured mice (Figure 5P), which indicated that PmTriTNE could act in synergy with the STING agonist to efficiently generate antitumor immune memory responses. To further demonstrate that PmTriTNE@CDA administration induced antitumor memory T cells, splenocytes obtained from the cured mice were transferred to NPG mice. We found that the transferred splenocytes prevented 4T1 tumorigenesis in vivo (Figure 5Q). In summary, PmTriTNE could activate T cells in the context of the TME, reverse PD‐L1 blockade to directly drive tumor cell lysis, and act synergistically with CDA to initiate an amplified immune cascade and thus achieve long‐term antitumor immunity against cold solid tumors.

### 2.6 PmTriTNE@CDA Showed Robust Antitumor Efficacy in Both Humanized CDX and PDX Mouse Models

**Figure 6:**
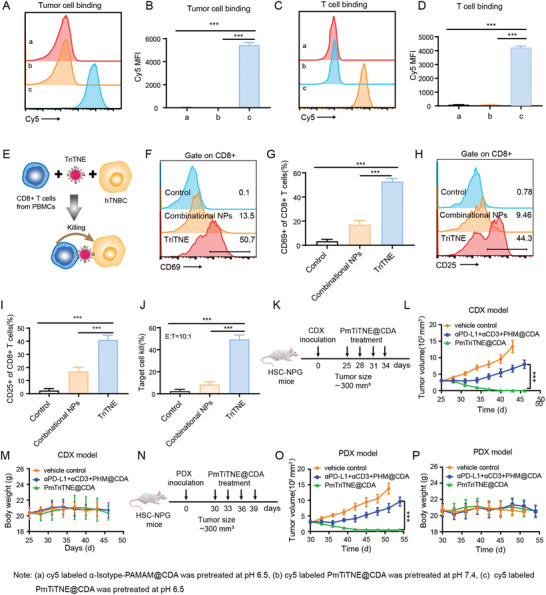
PmTriTNE@CDA showed potent antitumor efficacy. A–D) CD8+ T cells or MDA‐MB‐231 cells were incubated with cy5‐labeled *α*‐Isotype‐PAMAM@CDA pretreated with pH 6.5 buffer (i), cy5‐labeled PmTiTNE@CDA pretreated with pH 7.4 buffer (ii) or cy5‐labeled PmTiTNE@CDA pretreated with pH 6.5 buffer (iii) for 20 min at 4 °C. Then, Analyzed the specific binding of NPs to CD8+ T cells (A,B) or MDA‐MB‐231 cells (C,D) by flow cytometry. E) Schematic illustration of the co‐culture system used to evaluate the in vitro antitumor efficacy of TriTNE. F–I) CD8+ T cells were sorted from hPMBCs and then cocultured with MDA‐MB‐231 cells in the presence of *α*‐Isotype+*α*‐Isotype‐PAMAM (control), *α*‐CD3‐PAMAM + *α*‐PD‐L1‐PAMAM + HA‐PAMAM (combinational NPs) or TriTNE for 24 h. Then, CD69 (F,G) and CD25 (H,I) positive cells were analyzed by flow cytometry. J) Target cell lysis was evaluated at a 10:1 E:T ratio in the presence of TriTNE. K) Schematic illustration of the method used to evaluate the antitumor efficacy of PmTriTNE@CDA in the CDX model. MDA‐MB‐231 cells were inoculated into female human HSC‐engrafted NPG mice (HSC‐NPG) and treated with *α*‐Isotype+*α*‐Isotype‐PAMAM (control), *α*‐CD3 + *α*‐PD‐L1+ mPEG‐HA‐PAMAM@CDA (PHM@CDA) or PmTriTNE@CDA. L) Tumor volume and M) mouse body weight were monitored during the treatment period. N) Schematic illustration of the method used to evaluate the antitumor efficacy of PmTriTNE@CDA in the PDX model. PDX was inoculated into female HSC‐NPG mice carrying PDX and treated with *α*‐Isotype+*α*‐Isotype‐PAMAM (control), *α*‐CD3 + *α*‐PD‐L1+ mPEG‐HA‐PAMAM@CDA (PHM@CDA) or PmTriTNE@CDA (O,P). O) Tumor volume and P) mouse body weight were observed during the treatment period. Data are presented as the mean ± SD (For in vitro assays (A–J), *n* = 3. For in vivo assays (K–P), *n*  =  5). *p*‐values are calculated using one‐way ANOVA with Tukey's multiple comparison. *** *p* < 0.001.

It is critical to explore whether the use of PmTriTNE@CDA could be translated into the treatment of human TNBC (hTNBC). The modular design of PmTriTNE@CDA allows the easy exchange of existing therapeutic antibodies without sophisticated and time‐consuming protein engineering and modification, we fabricated PmTriTNE@CDA to target human CD3 and PD‐L1, evaluated their antitumor function in vitro and have shown that PmTriTNE@CDA pretreated at pH 6.5 could efficiently bind to CD8+ T cells and MDA‐MB‐231 cells, while PmTriTNE@CDA pretreated at pH 7.4 could not (**Figure** **6**A–D). The gating strategy of the MDA‐MB‐231 and T cells is as shown in Figures S15 and S16, Supporting Information. We further characterized TriTNE function in vitro and the schematic illustration of the in vitro assay as shown in Figure 6E. We found that TriTNE but not the combination of single specific NPs significantly increased CD69 (Figure 6F,G) and CD25 (Figure 6H,I) expression on CD8+ T cells. The gating strategy of the CD25 and CD69 on human CD8+ T cells is shown in Figure S17, Supporting Information. We further proved that TriTNE could redirect CD8+ T cells to kill MDA‐MB‐231 cells using the TDCC assay (Figure 6J). Thus, TriTNE could efficiently activate human CD8+ T cells and induce the cytotoxic killing of MDA‐MB‐231 cells. We subsequently evaluated the antitumor efficacy of TriTNE in an immune system humanized NOD/Prkdc^scid^/IL‐2R*γ* ^null^ (NPG) mouse model, in which human immune cells were reconstituted by CD34+ human hematopoietic stem cell (HSC) transplantation, as shown in Figure 6K. In this model, human HSCs reconstitute the human immune system in NPG mice and continuously produce various hematopoietic and immune cells, such as T cells, B cells, NK cells and myeloid cells. Moreover, immune cells develop de novo in mice and are tolerant to the mouse host. There is no graft versus host disease (GvHD), and the stable existence of human‐derived cells can still be detected after the model mice survive for one year.^[^ ^32^ ^]^ Thus, the HSC‐NPG model is the best model for exploring the antitumor efficacy of PmTriTNE@CDA. We found that treatment with PmTriTNE@CDA significantly inhibited tumor growth (Figure 6L) than the control treatment. Moreover, no overt toxicity or loss of body weight was observed during treatment (Figure 6M). Since over 90% of MDA‐MB‐231 cells are PD‐L1 and CD44 double‐positive cells (Figure S18, Supporting Information), to further explore whether PmTriTNE@CDA can overcome TNBC heterogeneity, we used the established TNBC PDX HSC‐NPG mouse model to evaluate the antitumor efficacy of PmTriTNE@CDA (Figure 6N). We found that PmTriTNE@CDA treatment could also efficiently control TNBC PDXs without inducing a loss of body weight during the treatment period (Figure 6O,P). These data indicated that PmTriTNE@CDA but not the combined treatment with single specific antibodies or single specific NPs could overcome the heterogeneity and immune therapy resistance of TNBC, which indicated that PmTriTNE@CDA gained novel functionality through the rational integration of multiple functionalities into one nanoplatform.

## 3 Discussion

TCEs can direct polyclonal T cells to kill poorly immunogenic tumors independent of the antigen specificity of the TCRs, which is promising for the targeting of poorly immunogenic tumors.^[^ ^33^ ^]^ However, the existing TCE strategy for TNBC is hindered by the “immunologically cold” TME, short half‐life, limited tumor enrichment, severe on‐target off‐tumor toxicity, inability to generate long‐term immune memory, and decreased activities against heterogeneous TNBC subpopulations.^[^ ^34^ ^]^ By taking advantage of nanotechnology, we rationally designed PmTriTNE@CDA to overcome TNBC heterogeneity and immunotherapy resistance.

The dense ECM acts as a physical barrier to limit the enrichment of nanomedicine within tumors and their penetration. TME‐responsive size‐tunable small NPs (<100 nm) can penetrate tumors better than conventional nanomedicines, which have a size of ≈100 nm.^[^ ^35^ ^]^ We chose PAMAM as a flexible platform to fabricate ultrasmall PmTriTNE, which were highly enriched in the tumor site and could efficiently mediate TDCC. STING signaling connects innate and adaptive immunity. STING agonists increased type I IFN expression to promote the maturation of DCs and thus effectively enabled the cross‐presentation of tumor antigens to activate T cells, increase TNBC immunogenicity, and recruit CD8+ T cells and NK cells into the tumor site to transform cold tumors into hot tumors. However, CDA is unstable in vivo, and the systemic administration of CDA causes serious toxicity. PmTriTNE could synergize with CDA to elicit immune memory without apparent toxicity upon systemic administration, which indicated that this strategy enables the application of new combinations that were previously considered impossible to achieve due to excessive toxicity.

CSCs have been identified across multiple solid cancers and play a key role in promoting intratumoral heterogeneity, immune evasion, target therapy resistance, and tumor recurrence.^[^ ^36^ ^]^ CSCs were found to be more enriched in TNBC tissues and cell lines than in tissues obtained from other breast cancer subtypes.^[^ ^30^ ^]^ CSCs in TNBC are characterized by repressed type I IFN signaling and reduced levels of antigen processing/presentation machinery (APM) and upregulated PD‐L1 to mediate immune evasion and immunotherapy resistance.^[^ ^3^, ^9^, ^37^ ^]^ Thus, the elimination of both non‐CSCs and CSCs is necessary to eradicate and cure TNBC. However, to the best of our knowledge, no TCEs can target heterogeneous TNBC populations. Moreover, because most TAAs are also expressed in normal tissues, the on‐target off‐tumor toxicity of TCE can be severe and even fatal. We used mPEG with a pH‐responsive linker as a mask to spatially shield TriTNE, which was largely intact in the systemic circulation, but preferentially unmasked TriTNE in the weakly acidic TME to minimize off‐target toxicity, which expands the targets for TAA selection. We chose CD44 and PD‐L1 as TAAs because they are overexpressed in TNBC and play key roles in CSC maintenance and immune escape.^[^ ^38^ ^]^ We selected an anti‐PD‐L1 antibody with a PD‐L1/PD‐1 interaction blockade function to target PD‐L1. The oligosaccharide HA, which shows a modest affinity for CD44, preferentially binds TNBC cells with high CD44 expression rather than CD44‐expressing normal cells. This dual CD44‐ and PD‐L1‐targeting strategy could improve tumor selectivity and binding affinity and effectively overcome TAA loss or PD‐L1‐mediated immune escape. Interestingly, we found that PmTriTNE and CDA exert a synergistic effect on reducing CSCs in vivo. Studies have found that the CSCs of TNBC exhibit higher CD44 and PD‐L1 expression than differentiated tumor cells. Moreover, CSCs with high metastatic potential are reportedly located at the leading edge of the tumor.^[^ ^39^ ^]^ IFN‐*β* can suppress CSC properties in TNBC.^[^ ^9a^ ^]^ Thus, we hypothesize that PmTriTNE@CDA efficiently eliminates CSCs by extrinsically targeting CD44‐ and PD‐L1‐high CSCs while intrinsically activating interferon signaling to repress CSCs.

In summary, by relying on a universally applicable TME‐responsive mPEG mask, PmTriTNE@CDA rationally integrates multispecific TCE, ICB and STING agonists to transform the cold TME into a hot TME, overcome TNBC heterogeneity by eliminating heterogeneous tumor cells, especially CSCs, and is well tolerated in vivo. These modularly designed nanoengagers could be readily applied to treat other types of “immunologically cold” cancers and pave the way for the development of new combinational immunotherapy strategies.

## 4 Experimental Section

### Preparation and Characterization of PmTriTNE@CDA

mPEG‐CHO (Mw = 5000) was purchased from Ponsure Biotechnology (F11098). Anti‐mouse CD3 antibodies, anti‐mouse PD‐L1 antibodies, anti‐human CD3 antibodies, and anti‐human PD‐L1 antibodies were purchased from Biolegend (100254, 124339, 317348, 329746). CDA (2′3′‐c‐di‐AM (PS)2 (Rp, Rp)) was purchased from InvivoGen (tlrl‐nacda2r‐01). Adipic acid dihydrazide (ADH), *N*‐(3‐dimethylaminopropyl)‐*N*'‐ethylcarbodiimide hydrochloride (EDC) and 1‐hydroxybenzotriazole (HOBT) were purchased from Aladdin Reagents. Generation 4 PAMAM dendrimer were obtained from Sigma‒Aldrich (163442‐67‐9). Enzymatically hydrolyzed prepared o‐HA (Mw = 2200) was obtained for Bloomage biotechnology. mPEG‐CHO (0.1 mmol) was dissolved in 15 mL of filtered purified water, and adipic acid dihydrazide (ADH) (0.2 mmol) was dissolved in 5 mL of filtered purified water. The two solutions were mixed and stirred at room temperature for 24 h. Then, the solution was rapidly dialyzed (MwCO = 3500) in 4 °C water. Portions of o‐HA (0.1 mmol) and EDC (0.2 mmol) were added to the solution. The pH of the mixture was adjusted to ≈6–7. Nitrogen was blown into the solution for 30 min with stirring. Then, the solution was sealed and stirred overnight. The small molecules were removed by dialysis (MwCO = 3500) at 4 °C in water. The solution was lyophilized to obtain mPEG‐HA. The mPEG‐HA (≈0.1 mmol), ɑ‐PD‐L1 (≈5 µmol), ɑ‐CD3 (≈5 µmol), EDC (0.2 mmol), and HOBT (0.2 mmol) powders were added to 20 mL of DMF and stirred until dissolved. Another 5 mL of DMF was used to dissolve PAMAM (5 µmol, dialyzed to remove methanol). The two solutions were mixed, sealed, and stirred for 5 h to obtain PmTriTNE. PmTriTNE (50 mg) and 6 mg CDA were dispersed in water and stirred for 1 h at room temperature to obtain CDA‐loaded PmTriTNE. One milliliter of CDA‐loaded PmTriTNE was sealed in a dialysis bag (MwCO = 7000) and immersed in 9 mL of PBS (pH 5.0, 6.5, and 7.4). The NPs were shaken at 37 °C in the dark and collected at predetermined time points for CDA quantification by high performance liquid chromatography (HPLC). A cumulative release curve was drawn to investigate the pH‐sensitive drug release behavior of the NPs. Cy5 NHS ester (AAT Bioquest, 146368‐14‐1) and cy7 NHS ester (AAT Bioquest, 477908‐53‐5) were used to label the PAMAM NPs. The total amount of antibody conjugated to the NPs was quantified by the Pierce Bicinchoninic Acid Protein Assay Kit (Fisher Scientific, 23225). The fluorescence intensity of FITC‐anti‐PD‐L1 antibody and APC‐anti‐CD3 antibody under a series of concentration gradients were measured by the microplate reader, respectively, and then the standard curves were plotted. The PmTriTNE were prepared with FITC‐anti‐PD‐L1 antibody and APC‐anti‐CD3 antibody. The fluorescence intensity of FITC and APC of the PmTriTNE was detected by a microplate reader (SpectraMax Paradigm, Molecular Devices, USA). Through calculation based on the respective standard curves, the ratio of anti‐PD‐L1, and anti‐CD‐3 antibodies in the PmTriTNE was determined.

### Cell Lines and Animals

The 4T1 and MDA‐MB‐231 cell lines were obtained from ATCC. Female BALB/c mice were obtained from Shanghai SLAC Laboratory Animal Co., Ltd. (Shanghai, China). Female NPG mice and female HSC‐NPG mice were obtained from Weida (Shanghai, China). Animal experiments were approved by the Ethics Committee of Renji Hospital, Shanghai Jiao Tong University School of Medicine and mouse strains were kept in specific‐pathogen‐free conditions and had free access to standard water and food in accordance with the institutional animal care and use guidelines of the Renji Hospital, Shanghai Jiao Tong University School of Medicine (SYXK(hu)2017‐0011).

### Human Samples

Tumor tissues and human PBMCs (hPBMCs) were obtained from the Yijishan Hospital of Wannan Medical College, and these experiments were approved by the Ethics Committee of the Yijishan Hospital of Wannan Medical College (LLSC‐2022‐124); written informed consent was obtained.

### Flow Cytometry Analysis of Immune Cells and CD8+ T‐Cell Sorting

Single‐cell suspensions were stained with Zombie Violet Fixable Viability dye (BioLegend) and then stained with antibodies. For intracellular cytokine and intranuclear transcription factor staining, the cells were fixed and permeabilized using the relevant buffer set (BioLegend, 421002, 424401). Cells were analyzed by FACSCelesta (BD Biosciences, USA). CD8+ T cells from mouse splenocytes or human PBMCs (hPBMCs) were sorted by a mouse CD8 or human CD8 negative selection kit (STEMCELL Technologies, 19853, 17953). The cell purity was over 90%. The antibodies used for the mouse immune cell experiment (BioLegend) were anti‐Foxp3 (320013), anti‐CD4 (100469), anti‐granzyme B (515403), anti‐CD25 (101907), anti‐CD8 (100753), anti‐CD69 (104514), anti‐CD80 (104732), anti‐CD86 (105027), anti‐CD3 (100205), anti‐CD19 (115503), anti‐CD49b (103521), anti‐CD45 (147707), anti‐PD‐L1 (124307), anti‐CD44 (103011), anti‐CD62L (104426), anti‐Ly6G (127603), anti‐Ki67 (151221), and anti‐CD11c (117307). Anti‐CD16/32 (101319) was used for Fc blocking. The antibodies used for the human immune cell experiment were as follows (from BioLegend): anti‐CD3 (317343), anti‐CD25 (356103), anti‐CD8 (344729), anti‐CD69 (310909), anti‐CD44 (103005), and anti‐PD‐L1 (329737). The gating strategy that was used to identify immune cell subsets was as follows: CD8+ T cells (CD45+CD3+CD8+); Tregs (CD45+CD3+CD4+Foxp3+); DCs (CD45+Dump (CD19, CD3, CD49b, Ly6G)‐CD11c+); and NK cells (CD45+CD3‐CD49b+).

### Binding Specificity of PmTriTNE@CDA In Vitro

CD8+ T cells or 4T1 cells were incubated with the indicated cy5‐labeled NPs for 20 min at 4 °C. The binding specificity of the NPs was measured by flow cytometry. To prevent nonspecific binding, cells were first blocked with 5% FBS in PBS.

### In Vitro CD8+ T‐Cell Activation and TDCC Assays

4T1 cells were seeded in a 96‐well plate and incubated overnight, followed by incubation with sorted CD8+ T cells at the indicated E:T ratio in the presence of *α*‐Isotype+*α*‐Isotype‐PAMAM NPs (containing 0.5 µg mL^−1^ *α*‐Isotype), *α*‐CD3+*α*‐PD‐L1+HA‐PAMAM NPs (containing 0.5 µg mL^−1^ *α*‐PD‐L1, 0.5 µg mL^−1^ *α*‐CD3), *α*‐CD3‐PAMAM NPs + *α*‐PD‐L1‐PAMAM NPs+ HA‐PAMAM NPs (containing 0.5 µg mL^−1^ *α*‐PD‐L1, 0.5 µg mL^−1^ *α*‐CD3), or TriTNE (containing 0.5 µg mL^−1^ *α*‐PD‐L1, 0.5 µg mL^−1^ *α*‐CD3) for 24 h. The expression of the T‐cell activation markers CD69 and CD25 was measured by flow cytometry. The IFN‐*γ* level in the supernatant was measured by ELISA (Biolegend, 430807). Target tumor cell killing was assessed after 24 h at 37 °C and 5% CO_2_ through the quantification of lactate dehydrogenase (LDH) release into cell supernatants by dead cells (Promega). Maximal lysis of target cells (100%) was achieved by incubation with Triton X‐100. Minimal lysis (0%) refers to target cells incubated with effector cells and *α*‐Isotype+*α*‐Isotype‐PAMAM. The percentage of TDCC was calculated as (sample release − spontaneous release)/(maximum release − spontaneous release) × 100.

### In Vitro Assessment of Immune Synapse (IS) Conjugate Formation

The CellTrace Violet (CTV) kit (Thermofisher, C34557) and CFSE kit (BioLegend, 423801) were used to label cells. CTV‐labeled CD8+ T cells were cocultured with CFSE‐labeled 4T1 cells in the presence of TriTNE for 30 min at 37 °C. Then, the T‐cell‐4T1 cell immune synapse conjugate was analyzed.

### In Vitro T‐Cell Tumor Infiltration Assays Using 3D Tumor Spheroid Models

A total of 2500 4T1 cells/well were seeded into a 96‐well U‐bottom ULA plate, centrifuged (125 × *g* 10 min) at RT and incubated for 3 days to form 3D spheroids. Then, CD8+ T cells and TriTNE were added. After 3 days of coculture, the 3D spheroids were washed and gently dissociated by Accutase (Gibco, 00‐4555‐56), and then the tumor‐infiltrated CD8+ T cells were measured by flow cytometry.

### Aldefluor Assay

4T1 cells or 4T1 tumor tissues were dissociated enzymatically to obtain a single‐cell suspension. An ALDEFLUOR kit (STEMCELL Technologies, 01700) was used for ALDH staining. The ALDH‐specific inhibitor diethylaminobenzaldehyde (DEAB)‐treated group was used as a control.

### Mammosphere Forming Assay

4T1 cells or 4T1 tumor tissues were dissociated enzymatically to obtain a single‐cell suspension and were seeded in ultralow attachment plates with 1500 cells per well. 4T1 Cells were cultured with mammary epithelial basal medium (MEMB) plus heparin (1 U mL^−1^), hydrocortisone (0.5 mg mL^−1^), insulin (5 mg mL^−1^), B‐27 (40 mg mL^−1^), epidermal growth factor (40 ng mL^−1^), fibroblast growth factor (40 ng mL^−1^, Peprotech, 100–18B), and methylcellulose (Sigma‒Aldrich, 9004‐67‐5) for 10 days. Mammospheres > 60 mm were counted.^[^ ^40^ ^]^

### Biocompatibility Assessment

BALB/c mice were intravenously injected with PmTriTNE@CDA twice a week. The mice were sacrificed 3 days after the last injection, and then, the following experiments were used to assess the biocompatibility of PmTriTNE: H&E staining of major organs; measurements of biochemical indicators (alanine transaminase [ALT] and aspartate transaminase [AST] levels) of liver function; and flow cytometry analysis of splenic T cells, B cells, myeloid cells, T_CM_ cells, and T_EM_ cells.

### Biodistribution of the NPs

Free cy7‐siR, cy7‐TriTNE@CDA and cy7‐PmTriTNE@CDA (containing 0.25 mg kg^−1^ *α*‐PD‐L1, 0.25 mg kg^−1^ *α*‐CD3) were injected intravenously. After 48 h, the in vitro fluorescence intensities of the tumors and major organs were measured using a fluorescence imager (Berthold Technologies, Germany).

### Pharmacokinetics

Free cy5, cy5‐TriTNE@CDA (containing 0.25 mg kg^−1^ *α*‐PD‐L1, 0.25 mg kg^−1^ *α*‐CD3) or cy5‐PmTriTNE@CDA (containing 0.25 mg kg^−1^ *α*‐PD‐L1, 0.25 mg kg^−1^ *α*‐CD3) was injected intravenously into SD rats. Plasma cy5 concentrations were measured with a microplate reader (Thermo Scientific, USA) at 0, 0.25, 0.5, 1, 2, 4, 6, 8, 12, 24, and 48 h after administration.

### A 4T1 Tumor Model

A total of 5×10^5^ 4T1 cells mixed with Matrigel (Corning Life Sciences, 354262) was inoculated into the fourth mammary fat pad of syngeneic BALB/c mice to generate the orthotopic TNBC tumor model. Orthotopic TNBC tumor model was established by the inoculation of 5×10^5^ 4T1 cells mixed with Matrigel into the fourth mammary fat pad of BALB/c mice. After the tumors grew to 250–350 mm^3^ (mice with small tumors were excluded from the experiments), the tumor‐bearing mice were randomly grouped and intravenously administered *α*‐Isotype+*α*‐Isotype‐PAMAM (control), ɑ‐PD‐L1+ ɑ‐CD3+mPEG‐HA‐PAMAM (containing 0.25 mg kg^−1^ *α*‐PD‐L1, 0.25 mg kg^−1^ *α*‐CD3), PmTriTNE (containing 0.25 mg kg^−1^ *α*‐PD‐L1, 0.25 mg kg^−1^ *α*‐CD3), ɑ‐PD‐L1+ɑ‐CD3+mPEG‐HA‐PAMAM@CDA (containing 0.25 mg kg^−1^ *α*‐PD‐L1, 0.25 mg kg^−1^ *α*‐CD3, and 0.06 mg kg^−1^ CDA), or PmTriTNE@CDA (containing 0.25 mg kg^−1^ *α*‐PD‐L1, 0.25 mg kg^−1^ *α*‐CD3 and 0.06 mg kg^−1^ CDA) every 3 days 4 times. To assess the health of the mice, body weight, general activity, and physical appearance were monitored during treatment. The mice were sacrificed when the tumor volume reached 1500 mm^3^ for animal welfare. Tumor growth curves were calculated according to the following formula: width^2^ × length × 0.5.

### Evaluation of the Protective Immune Memory Response After Tumor Clearance In Vivo

The PmTriTNE@CDA‐cured BALB/c mice after treatment were rechallenged with 4T1 tumor cells (5 × 10^5^) at 50 days after primary tumor injection using untreated naïve mice as controls. Then, tumor growth was monitored. To more directly confirm the existence of memory T cells, a total of 2 × 10^7^ splenocytes from PmTriTNE@CDA cured mice were adoptively transferred to NPG mice. Then, 5 × 10^5^ 4T1 cells were inoculated into the fourth mammary fat pad of NPG mice the next day and tumor growth was monitored.

### Characterization of the Intratumor Immune Response

Tumors were gently disrupted and then digested by collagenase/hyaluronidase (Sigma‐Aldrich, H4272, C2674). Percoll gradient centrifugation was applied to obtain the TILs. Then, the TILs were analyzed by flow cytometry. The mRNA expression levels of ISGs, including CXCL9 and IFN‐*β*, were analyzed by quantitative PCR with reverse transcription (RT‐qPCR).

### Humanized Xenograft Models and Therapeutic Efficacy Evaluation

HSC‐NPG mice were used to establish CDX and PDX models. One hundred days after HSC transplantation, the HSC‐NPG mice were examined to assess the percentages of circulating human CD45+ cells of HSC‐NPG, and HSC‐NPG mice >30% hCD45+ in peripheral blood were used for the CDX and PDX models. A total of 1 × 10^6^ MAD‐MB‐231 cells mixed with Matrigel (Corning Life Sciences) were inoculated into the fourth mammary fat pad of HSC‐NPG mice to generate the orthotopic CDX tumor model. HSC‐NPG mice were orthotopically implanted with ≈3 × 3 mm tumor fragments to generate the PDX tumor model. Once tumors reached 250–350 mm^3^, animals were randomized and treated with intravenously administered *α*‐Isotype+*α*‐Isotype‐PAMAM (control) (containing 0.5 mg kg^−1^ *α*‐isotype), *α*‐CD3 + *α*‐PD‐L1+ mPEG‐HA‐PAMAM@CDA (PHM@CDA) (containing 0.25 mg kg^−1^ *α*‐ human PD‐L1, 0.25 mg kg^−1^ *α*‐human CD3, and 0.06 mg kg^−1^ CDA), or PmTriTNE@CDA (containing 0.25 mg kg^−1^ *α*‐ human PD‐L1, 0.25 mg kg^−1^ *α*‐ human CD3, and 0.06 mg kg^−1^ CDA) every 3 days 4 times. To assess the health of the mice, body weight, general activity, and physical appearance were monitored during treatment. The mice were sacrificed when the tumor volume reached 1500 mm^3^ for animal welfare. Tumor growth curves were calculated according to the following formula: width^2^ × length × 0.5.

### Statistical Analysis

The data were analyzed with GraphPad Prism 8.0 software. The data distributions of values were checked for Gaussian distribution. For normally distributed data, student's *t*‐test was used for comparisons of 2 groups and one‐way ANOVA with Tukey's multiple comparison post‐hoc test was used for comparisons of more than 2 groups. For all the in vitro experiments, *n* = 3 (3 biologically independent reduplicates). For all the in vivo experiments, *n* = 5 (5 mice per groups). Data are shown as the mean ± SD. Significance was defined as *p* ≤ 0.05. Statistical significance is indicated by *p < 0.05, **p < 0.01, and ***p < 0.001.

## Conflict of Interest

The authors declare no conflict of interest.

## Supporting information

Supporting Information

## Data Availability

The data that support the findings of this study are available from the corresponding author upon reasonable request.
