# Supplementary material for: Systemic Delivery of mPEG‐Masked Trispecific T‐Cell Nanoengagers in Synergy with STING Agonists Overcomes Immunotherapy Resistance in TNBC and Generates a Vaccination Effect
Source: Adv Sci (Weinh). 2022 Sep 11;9(32):2203523. doi: 10.1002/advs.202203523 (PMC9661824; doi:10.1002/advs.202203523)
Supplement: Supplementary file 1 — Supporting Information [file ADVS-9-2203523-s001.pdf]

Systemic Delivery of mPEG-masked Trispecific T-cell Nanoengagers in Synergy with STING Agonists Overcomes Immunotherapy Resistance in TNBC and Generates a Vaccination Effect

Ming Shen[a,c#], Chuanrong Chen[a,d#], Qianqian Guo[b,], Quan Wang[b], Jinghan Liao[a], Liting Wang[b], Jian Yu[a], Man Xue[c], Yourong Duan[a*], Jiali Zhang[a*]

[a]State Key Laboratory of Oncogenes and Related Genes, Shanghai Cancer Institute, Renji Hospital, School of Medicine, Shanghai Jiao Tong University, Shanghai,200032, China

[b]State Key Laboratory of Oncogenes and Related Genes, Renji Hospital, School of Biomedical Engineering, Shanghai Jiao Tong University, Shanghai, 200127, China

[c]Shanghai Institute for Biomedical and Pharmaceutical Technologies, Shanghai, 200032, China

[d]Department of Oncology, Yijishan Hospital of Wannan Medical College, Wuhu, 240001, China

[#]These authors contributed equally to this work.

[*]Corresponding Authors

E-mail：jlzhang@shsci.org ; yrduan@shsci.org

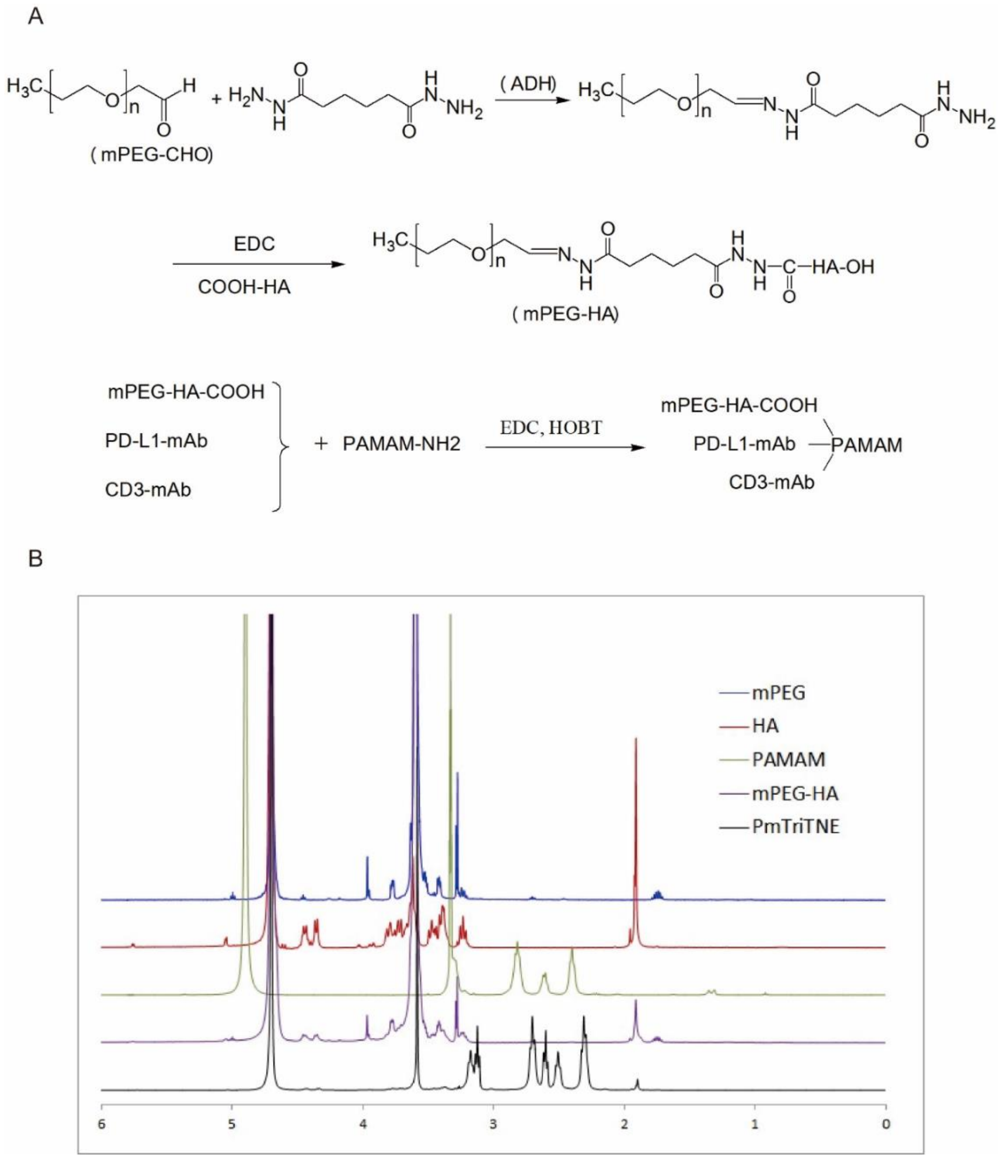

Figure S1. The synthetic route and characterization of PmTriTNE. (A) The synthetic route of PmTriTNE. The mPEG and o-HA were linked via a hydrazone bond to construct mPEG-HA. The mPEG-HA, α-PD-L1 and α-CD3 were attached to PAMAM by amidation reaction. (B) The ^{1}H NMR spectra of PmTriTNE. The characteristic peaks of the materials were reflected in the spectrum of PmTriTNE.

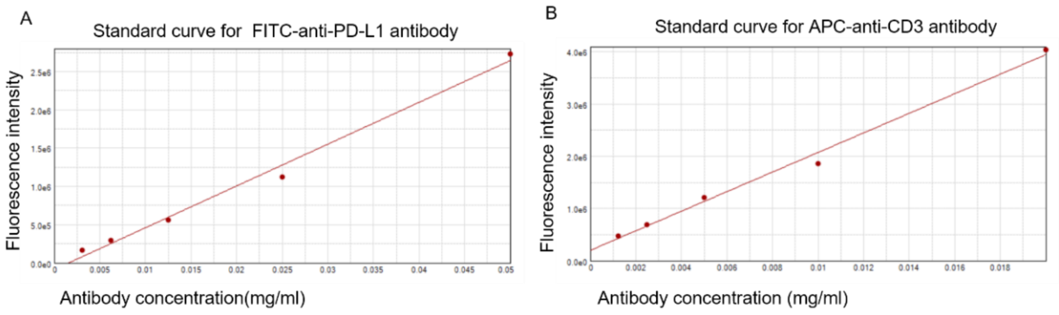

Figure S2. A. The standard curve for FITC-anti-PD-L1 antibody. B. The standard curve for APC-

anti-CD3 antibody.

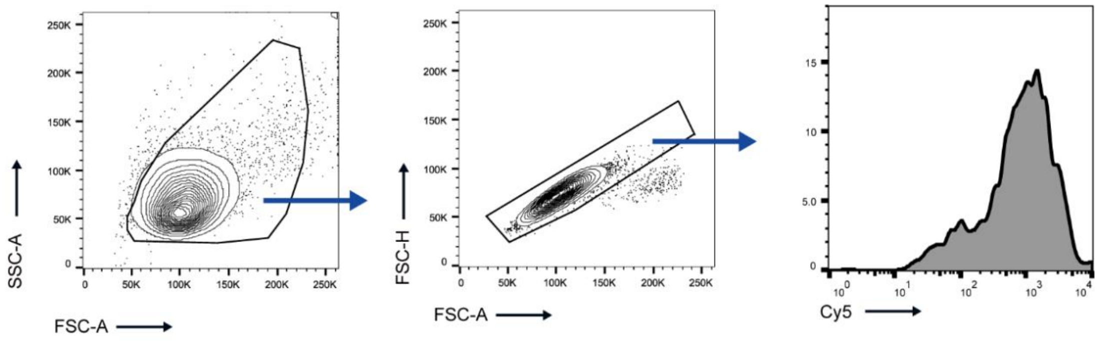

Figure S3. The gating strategy of the for the 4T1 cells binding.

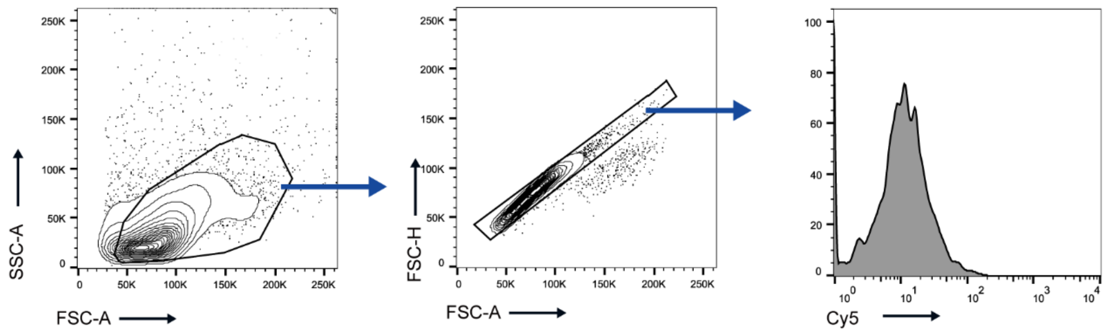

Figure S4. The gating strategy of the for the T cells binding.

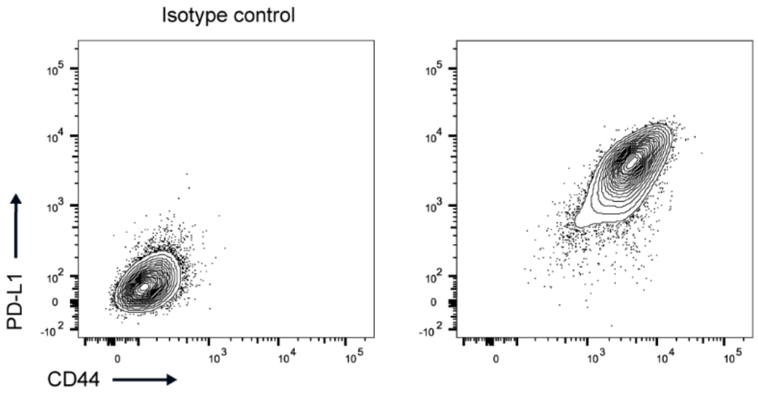

Figure S5. Flow cytometric analysis of PD-L1 and CD44 expression on 4T1 cells surface.

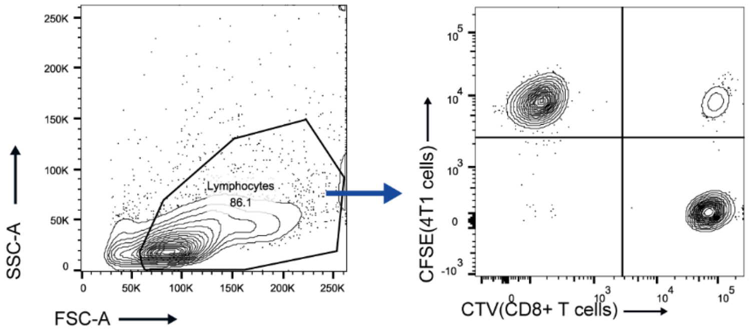

Figure S6. The gating strategy of the immune synapse conjugate formation (CFSE+CTV+) population

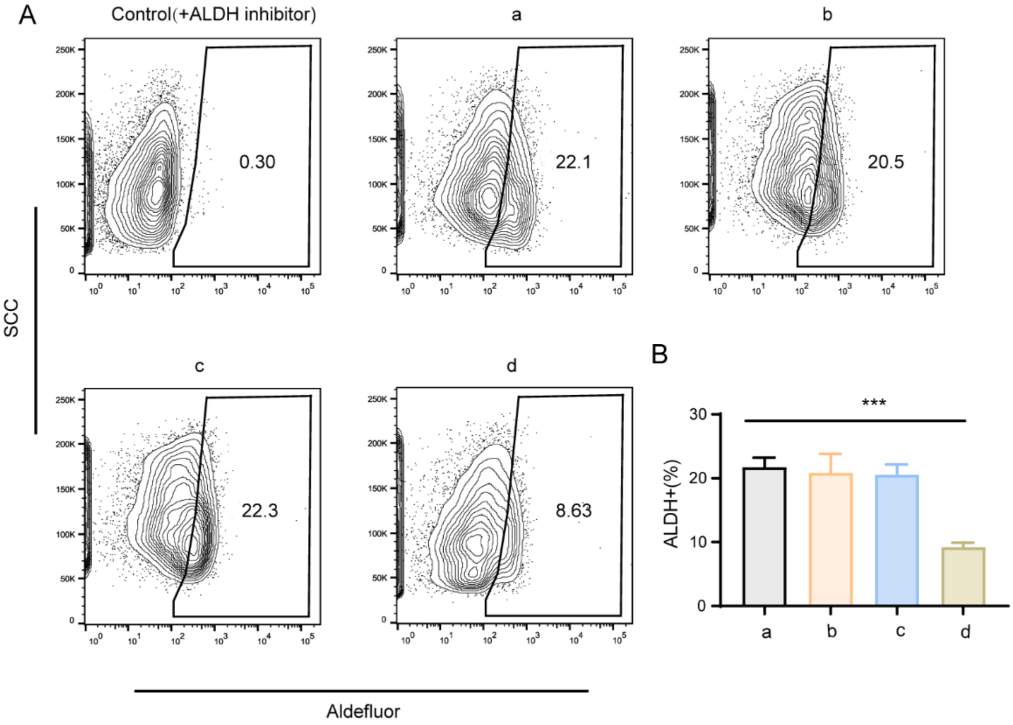

Figure S7. The 4T1 were cultured for 1 day, and then CD8+ T cells (E/T ratio = 10:1) were added in the presence of the α-Isotype+α-Isotype-PAMAM(a), α-CD3+α-PD-L1+HA-PAMAM(b), α-CD3-PAMAM + α-PD-L1-PAMAM + HA-PAMAM (c) or TriTNE (d) for 24 h. (A) Flow cytometry analysis of percentage of ALDH+ 4T1 cells in vitro. (B) Statistical analysis of percentage of ALDH+ 4T1 cells from A.

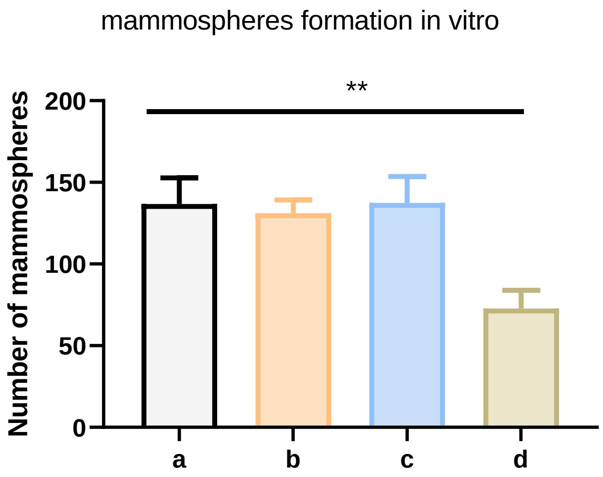

Figure S8. Mammosphere forming assay in vitro. The 4T1 were cultured for 1 day, and then CD8+ T cells (E/T ratio = 10:1) were added in the presence of the α-Isotype+α-Isotype-PAMAM(a), α-CD3+α-PD-L1+HA-PAMAM(b), α-CD3-PAMAM + α-PD-L1-PAMAM + HA-PAMAM (c) or TriTNE(d) for 24 h. then 4T1 cells were collected (CD8+ cells were depleted by magnet beads) and plated to perform the in vitro sphere-forming assay.

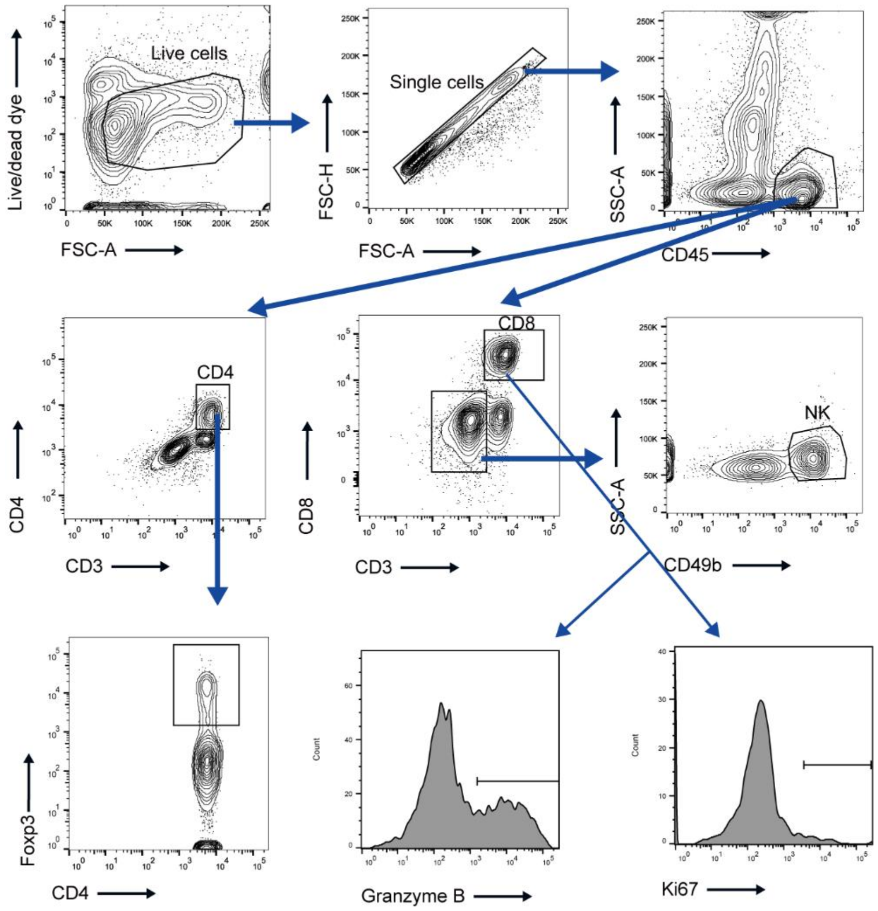

Figure S9. Gating strategy for tumor infiltrating lymphocytes (TILs).

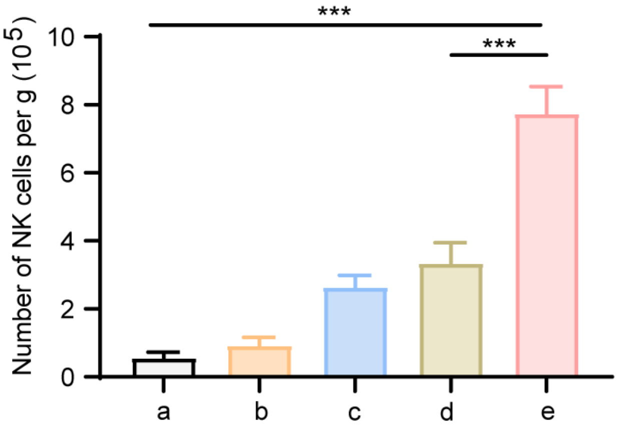

Figure S10. The absolute number of tumor infiltrated NK cells. α-Isotype+α-Isotype-PAMAM NPs (control) (a), α-PD-L1+α-CD3+mPEG-HA-PAMAM (b), PmTiTNE (c), α-PD-L1+α-CD3+mPEG-HA-PAMAM@CDA (d) or PmTiTNE@CDA (e).

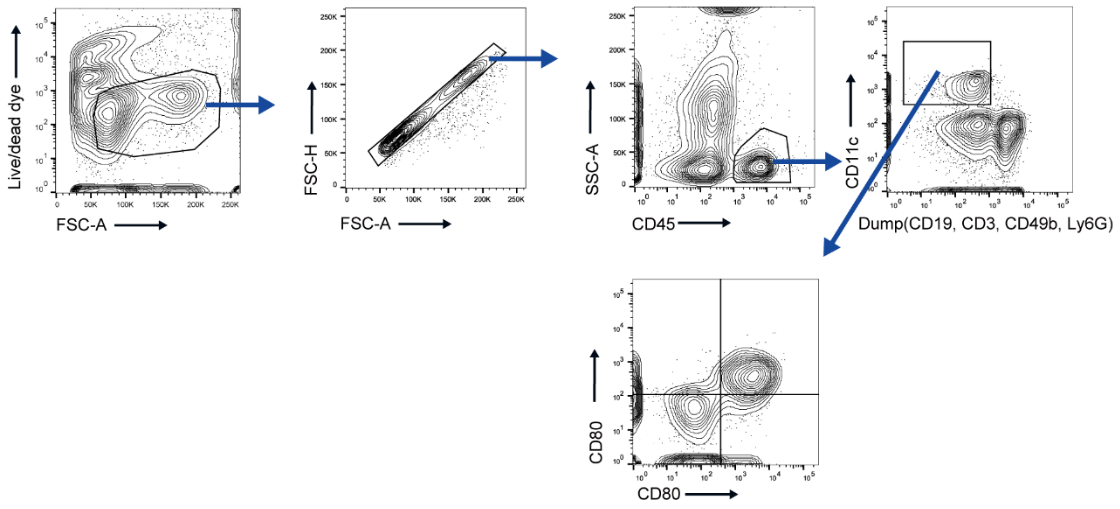

Figure S11. The gating strategy of the CD80 and CD86 on DCs

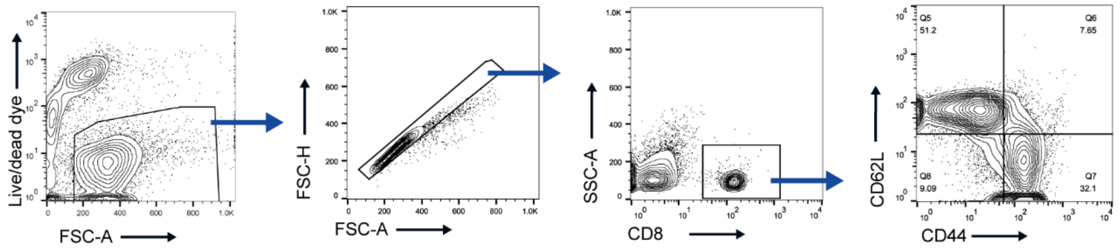

Figure S12. The gating strategy of the CD8+ T cells in TDLN.

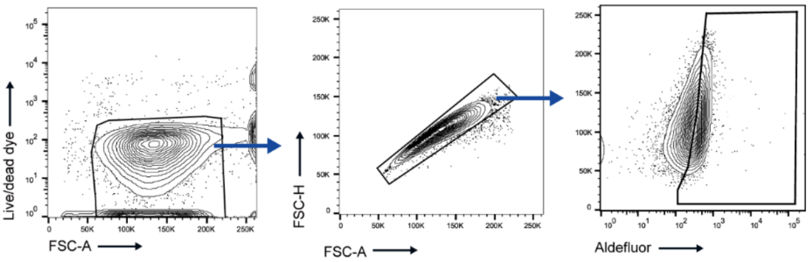

Figure S13. The gating strategy of ALDH+ 4T1 cells.

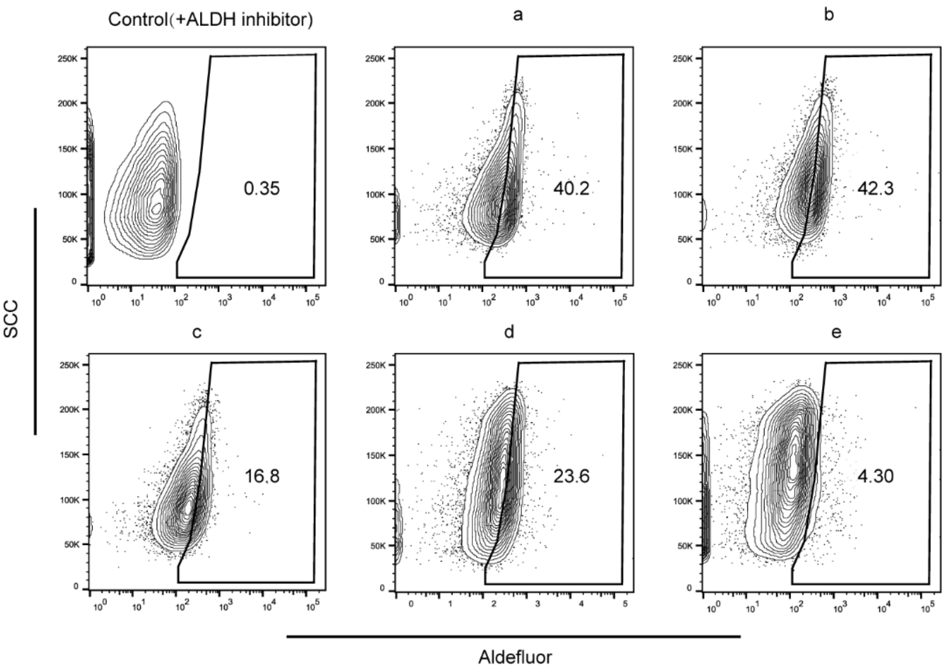

Figure S14. Flow cytometry analysis of percentage of ALDH+ 4T1 cells in vivo. α-Isotype+α-Isotype-PAMAM NPs (control) (a), α-PD-L1+α-CD3+mPEG-HA-PAMAM (b), PmTiTNE (c), α-PD-L1+α-CD3+mPEG-HA-PAMAM @CDA (d) or PmTiTNE@CDA (e).

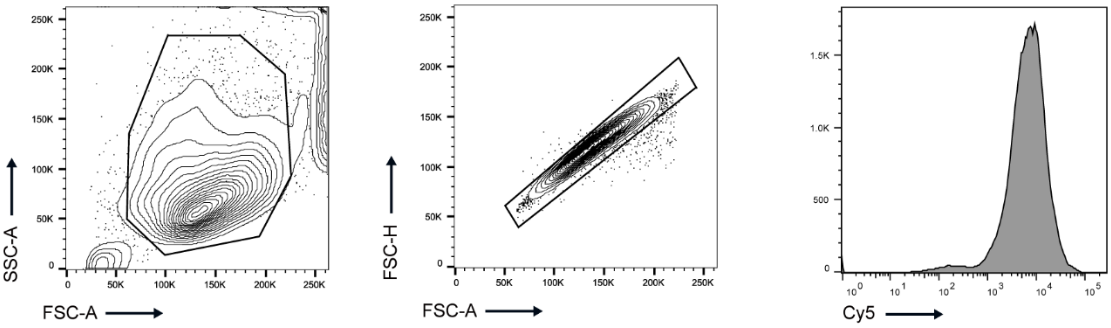

Figure S15. The gating strategy of the for the MDA-MB-231 cells binding.

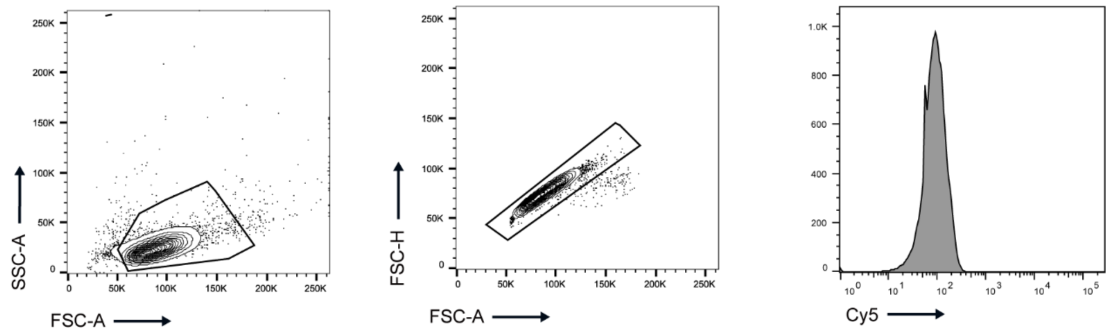

Figure S16. The gating strategy of the for the human T cells binding.

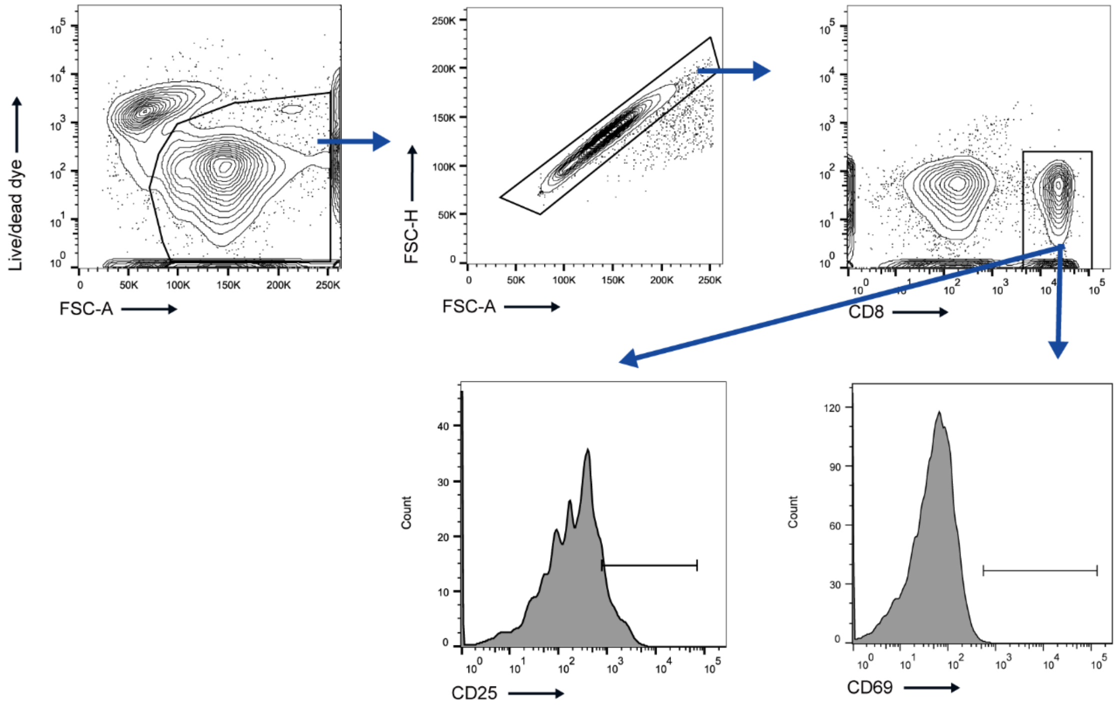

Figure S17. The gating strategy of the CD25 and CD69 on human CD8+ T cells.

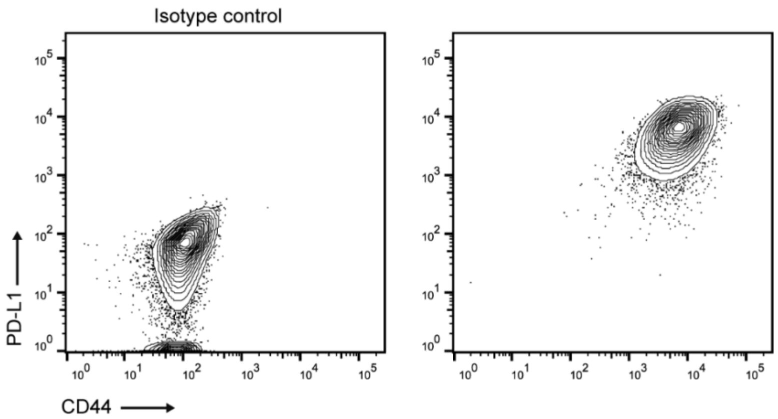

Figure S18. Flow cytometric analysis of PD-L1 and CD44 expression on MDA-MB-231 cells surface.

Abbreviation list

ADH	adipic acid dihydrazide
ALDH	aldehyde dehydrogenase
ALT	alanine aminotransferase
AST	aspartate aminotransferase
BiTEs	bispecific T-cell engagers
CDA	c-di-AMP
CDNs	cyclic dinucleotides
CDX	cell line-derived xenograft
CSC	cancer stem cells
DCs	dendritic cells
DEAB	diethylaminobenzaldehyde
EDC	N-(3-dimethylaminopropyl)-N'-ethylcarbodiimide hydrochloride
EPR	enhanced penetration residence
HOBT	1-Hydroxybenzotriazole
HPLC	High Performance Liquid Chromatography
Hyd	hydrazone bond
ICB	immune checkpoint blockade
ISGs	interferon-stimulated genes
iRAEs	immune-related adverse events
IS	immune synapse
LDH	lactate dehydrogenase
NPs	nanoparticles
o-HA	oligomeric hyaluronic acid
PDX	patient-derived xenograft
PmTriTNE@CDA	CD44×PD-L1/CD3 trispecific T-cell nanoengager loaded with CDA
STING	The stimulator of interferon genes
TAAs	tumor-associated antigens
TCE	T-cell engagers

TCR	T-cell receptors
TCM	central memory T cells
TDCC	T-cell–dependent cellular cytotoxicity
TDLNs	tumor drain lymph nodes
TEM	effect memory T cells
TILs	tumor-infiltrating lymphocytes
TME	tumor microenvironment
TNBC	triple-negative breast cancer
Tregs	regulatory T cells
TriTNE	CD44×PD-L1/CD3 trispecific T-cell nanoengager
